# Supplementary material for: Dimerization regulates both deaminase-dependent and deaminase-independent HIV-1 restriction by APOBEC3G
Source: Nat Commun. 2017 Sep 19;8:597. doi: 10.1038/s41467-017-00501-y (PMC5605669; doi:10.1038/s41467-017-00501-y)
Supplement: Supplementary file 1 — Supplementary Information [file 41467_2017_501_MOESM1_ESM.pdf]

File Name: Supplementary Information
Description: Supplementary Figures

File Name: Peer Review File
Description:

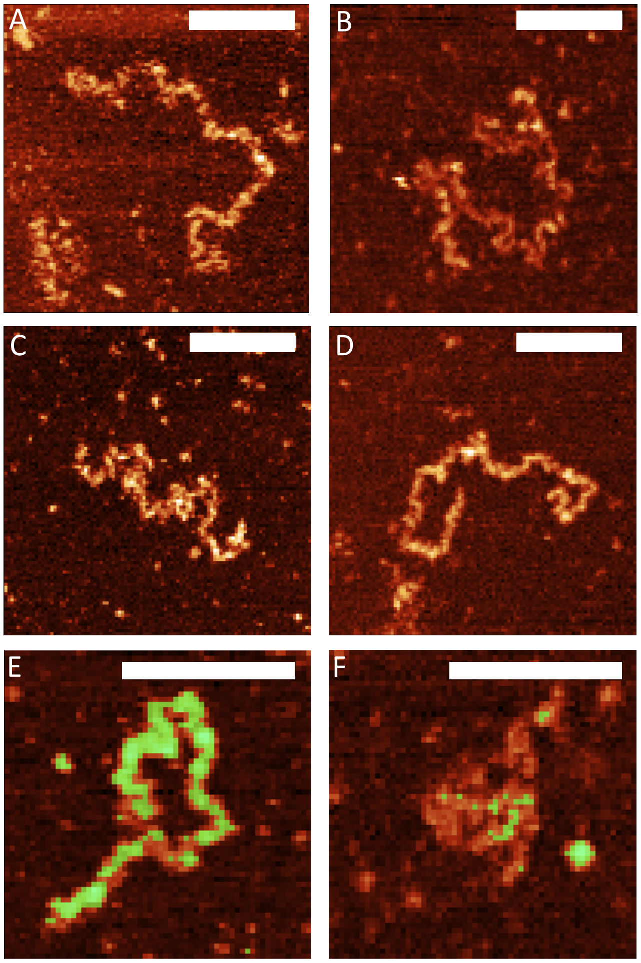

Supplementary Figure 1: Sample AFM image of linearized m13 ssDNA imaged in liquid on a mica surface after incubation with WT (A), IY (B), RDDQ (C), or FW (D) mutant A3G. During incubation A3G binds to the ssDNA, and can be identified by the increase in image height as seen by the AFM cantilever along the contour length of the ssDNA (shown as bright white spots in the image). Using a height threshold of 4 nm, individual bound protein clusters are identified, marked in green for WT (E) and FW (F). The total integrated volume of each binding location is calculated to estimate the number of A3G subunits per cluster in order to measure oligomerization activity. Scale bars are 200 nm.

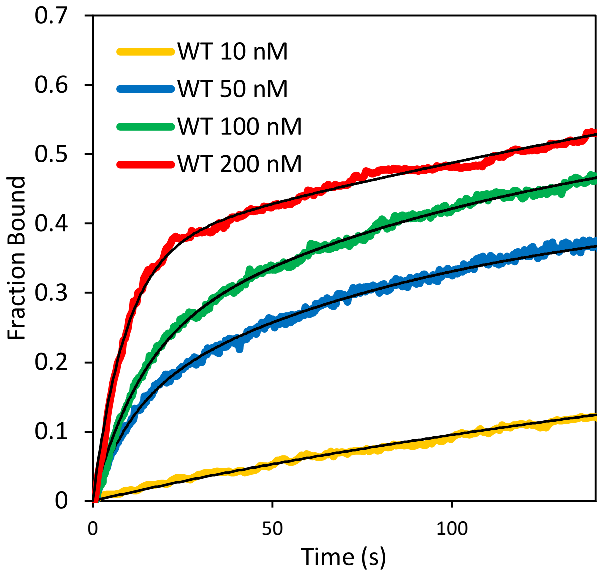

c	k_{fast} (s)	k_{slow} (s)
**10 nM**	-	0.00363
**50 nM**	0.0561	0.00783
**100 nM**	0.121	0.00840
**200 nM**	0.214	0.00729

Supplementary Figure 2: Fractional binding over time for ssDNA incubated with varying concentrations of WT A3G. Every experimental curve is fit (black lines) using the two-step binding model (Eq. 2). This returns two exponential rates (k_{fast} and k_{slow}), which are reported for the sample curves. At low concentrations (10 nM) the ssDNA is not saturated by the binding of A3G monomers and the rate of oligomer formation is greatly reduced. Note that the 10 nM data presented here is for illustrative purposes only and this data is not used to determine the fundamental rate constants in the manuscript as k_{fast} is not well defined. In contrast, for high A3G concentrations (>50 nM), while the initial binding rate of free A3G is concentration dependent, the rate of oligomer formation is relatively constant as A3G monomers saturate the ssDNA substrate as $ck_1 >> k_{-1}$.

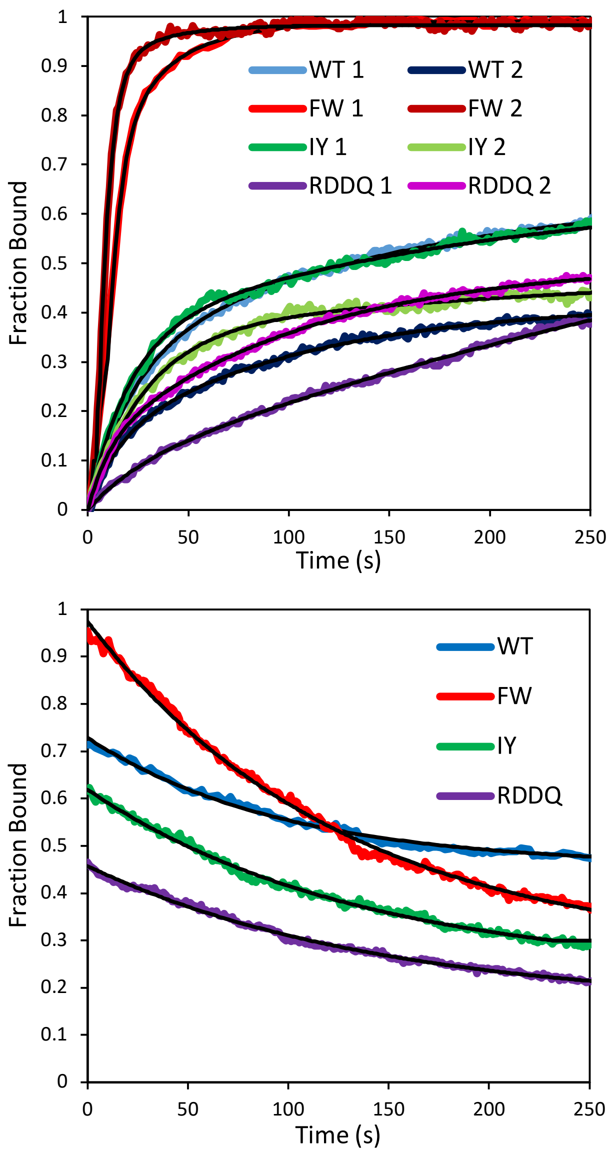

Supplementary Figure 3: (a) Sample binding curves for ssDNA incubated with 50 nM WT (blue), FW (red), IY (green), and RDDQ (purple) mutant A3G fit with two-exponential function (black lines) as described in main text. The NTD mutant (FW) A3G quickly and fully saturates the ssDNA. In contrast, WT and CTD mutant (IY and RDDQ) A3G shows slower initial binding and significant binding over long timescales due to dimerization. Additionally, the final degree of binding saturation is highly variable, suggesting a stochastic process (likely dimerization) inhibits fully saturated binding. **(b)** Sample dissociation curves for ssDNA incubated with 50 nM WT (blue), FW (red), IY (green), and RDDQ (purple) mutant A3G fit with single exponential function (black lines) as described in main text. Some fraction of bound A3G does not dissociate, depending on A3G variant and incubation time. The dissociable fraction decays at a single rate (k_{-1}) on the timescale of ~100 s for all A3G variants.

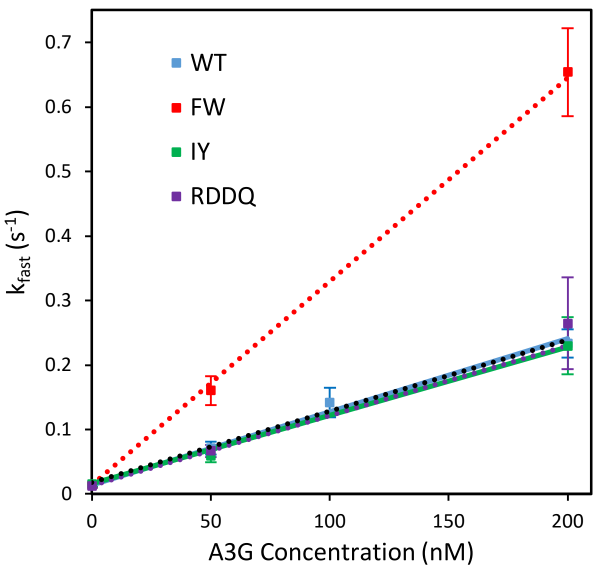

Supplementary Figure 4: Concentration dependence of k_{fast} for WT (blue), FW (red), IY (green), and RDDQ (purple) mutant A3G. Error bars are standard error of the mean. Data points at concentration zero are derived from fitting a single exponential function to dissociation curves (Fig. 3b) while all other data points are derived from fitting two exponential function to binding curves (Fig. 3a). Lines of corresponding color are plotted using the values of k_{-1} (y-intercept) and k_1 (slope) derived in the manuscript to show the expected concentration dependence of k_{fast}. Dotted black line is a direct weighted, linear fit of WT data points and produces nearly identical results to the derived values k_{-1} and k_1. Some lines are solid or dotted purely for enhanced visibility of overlapping lines. In contrast to k_{fast}, k_{slow} does not differ significantly over the range of concentrations measured (50-200 nM).

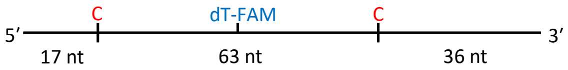

Labeled DNA Sequence (118 nt)
GAA TAT ATG TTG AGA CCC AAA GTA ATG AGA
GAT TGA (dT-FAM)TA GAT GAG TGT AAT GTG ATA
TAT GTG TAT GAA AGA TAT AAG ACC CAA AGA
GTA AAG TTG TTA ATG TGT GTA GAT ATG TTA A

Unlabeled DNA Sequence (69 nt)
AAA GAG AAA GTA ATA AGG AAA GAG TAA AGT
ATA ATC AAA TAA ACA ATC ATT CTA CAC ATT CAT
ACA ATT

Supplementary Figure 5: Labeled DNA construct used in deamination assay. The third cytidine (red) of the CCC motif is targeted for deamination by A3G. Deamination and cleaving at the 5′, 3′, or both sites will result in a labeled construct 100, 81, or 63 nts in length, respectively.
